# GNA12 regulates C5a-induced migration by downregulating C5aR1-PLCβ2-PI3K-AKT-ERK1/2 signaling

**DOI:** 10.52601/bpr.2023.230001

**Published:** 2023-02-28

**Authors:** Haonan Yu, Zhihua Liu

**Affiliations:** 1 Key Laboratory of Infection and Immunity, Institute of Biophysics, Chinese Academy of Sciences, Beijing 100101, China; 2 University of Chinese Academy of Sciences, Beijing 100049, China; 3 Institute for Immunology, Tsinghua University, Beijing 100084, China; 4 Tsinghua-Peking Center for Life Sciences, Beijing 100084, China

**Keywords:** Inflammatory bowel disease, GNA12, Macrophages, Chemotactic migration

## Abstract

*Gna12* has been identified as one of the reported inflammatory bowel disease (IBD) susceptibility genes in genome-wide association studies (GWAS). However, the function of GNA12 in intestinal homeostasis remains unknown. Here we report that GNA12, a G-protein α subunit, regulates C5a-induced migration in macrophages. Deficiency of GNA12 results in enhanced migration induced by C5a in macrophages. Mechanistically, GNA12 suppresses C5a-induced migration by downregulating the C5aR1-PLCβ2-PI3K-AKT-ERK1/2 signaling. Therefore, our study reveals that GNA12 is an anti-inflammatory factor, which might alleviate the development of inflammation by inhibiting the excessive chemotactic migration of macrophages.

## INTRODUCTION

Inflammatory bowel disease (IBD) is a chronic inflammatory disease of the gastrointestinal tract, including two main forms: Crohn’s disease (CD) and ulcerative colitis (UC) (Szigethy *et al*. 2010). Both genetic and complex environmental factors contribute to the etiology and pathogenesis of IBD (Kaser *et al*. 2010; Monteleone *et al*. 2006). Genome-wide association studies (GWAS) have identified multiple genetic loci associated with IBD susceptibility, however, the mechanisms underlying many of these associations remain to be uncovered. The gene encoding guanine nucleotide-binding protein subunit alpha-12 (GNA12) has been reported to harbor an IBD-susceptibility locus (Anderson *et al*. 2011).

GNA12 belongs to the G_12_ family of G-protein α subunits. The G_12_ family is composed of GNA12 and GNA13 (Strathmann and Simon 1991). GNA12/13-mediated signaling pathways are involved in a variety of physiological processes, including cell growth, cell migration, angiogenesis, platelet activation and apoptosis (Arthofer *et al*. 2016; Grzelinski *et al*. 2010; Joshi *et al*. 2015; Moers *et al*. 2003; Yanamadala *et al*. 2007). It is largely believed that GNA12 and GNA13 may functionally overlap in these processes. At the same time, it has been indicated that GNA12 and GNA13 have distinct functions. For example, mutations in GNA13, but not GNA12, have been described as the top five frequently occurring mutations in tumors of B-cell origins such as diffuse large cell B-cell lymphomas (DLBCL) (Dobashi 2016). However, GNA12, but not GNA13, is critically important for fibroblast migration and the development of pulmonary fibrosis through specifically interacting with ARAF to activate ERK signaling pathway (Gan *et al*. 2012, 2013). In addition, SNP (rs798502) in *Gna12* is associated with IBD susceptibility, which suggests that GNA12 may have distinct roles in maintaining intestinal homeostasis (Anderson *et al*. 2011; McCole 2014).

Complement component 5a (C5a) is cleaved enzymatically from its precursor, C5, upon activation of the complement cascade. C5a has a potent proinflammatory activity, recruiting myeloid cells such as neutrophils, eosinophils, monocytes and macrophages to the site of infection (Guo and Ward 2005; Marder *et al*. 1985). There are two receptors for C5a, C5aR1 and C5aR2. C5a exerts its pro-inflammatory activity mainly through binding to complement 5a receptor 1, C5aR1 (also known as CD88) (Guo and Ward 2005). C5aR2 largely remains an enigmatic receptor for C5a with no clearly defined role in host defense (Zhang *et al*. 2017). By binding to C5aR1, C5a results in the phosphorylation of p38 MAPK and ERK1/2 through C5aR1-p38 and C5aR1-PLCβ2-PI3K-AKT-ERK1/2 signaling pathways, which can induce chemotactic migration in macrophages (Chiou *et al*. 2004). Previous studies have shown that the content of C5a in murine colon tissue is significantly increased in colitis (Jain *et al*. 2013). Blocking C5a action either by knockout of C5aR1 or by treatment of C5a receptor antagonist PMX205 alleviates DSS-induced colitis in mice (Jain *et al*. 2013; Johswich *et al*. 2009).

Here we found that GNA12 suppressed C5a-induced migration in macrophages. Mechanistically, GNA12 inhibited C5a-induced migration in macrophages by inhibiting the C5aR1-PLCβ2-PI3K-AKT-ERK1/2 signaling pathway. Finally, we determined that GNA12 interacted with C5aR1 and PLCβ2. Collectively, our data demonstrate that GNA12 regulates C5a-induced migration in macrophages through a C5aR1-PLCβ2-PI3K-AKT-ERK1/2 axis.

## RESULTS

### GNA12 is expressed in murine macrophages

**Figure 1:**
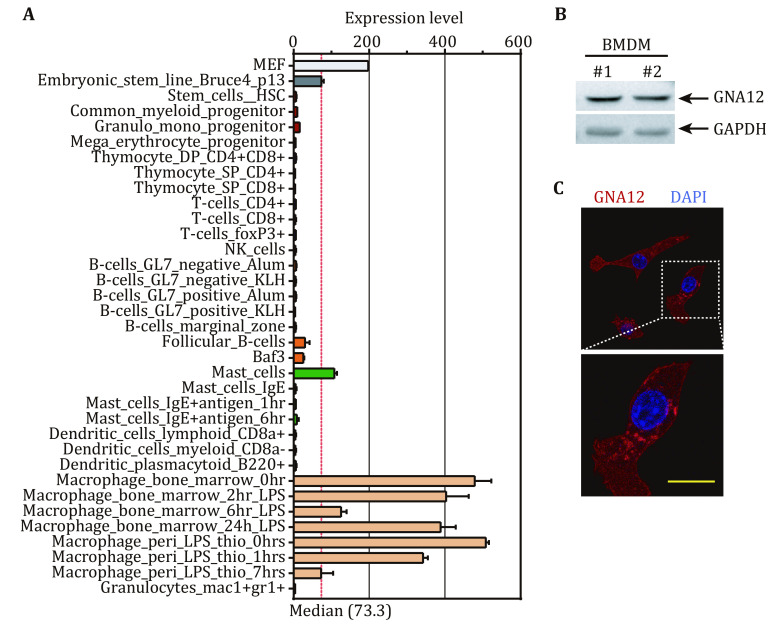
Murine macrophages highly express GNA12. **A** Relative expression of *Gna12* in murine cells in BIOGPS database. **B** Immunoblot of GNA12 in WT murine bone marrow-derived macrophages (BMDMs). GAPDH was used as a loading control. BMDMs from 2 mice (#1 and #2) were detected. **C** Confocal images of GNA12 (red) in *Gna12*^*mCherry*^ RAW264.7 cells. Nuclei were counterstained with Hoechst (blue). Boxed areas are shown at higher magnification in the panels below. Scale bar, 10 μm

BIOGPS database indicates *Gna12* mainly expresses in bone marrow-derived macrophages (BMDMs) and peritoneal macrophages (Fig. 1A). We confirmed the expression of GNA12 in BMDMs using immunoblotting (Fig. 1B).

The lack of GNA12 antibodies suitable for immunostaining precluded us from examining the cellular localization of endogenous GNA12. To determine the cellular localization of GNA12 in macrophages, we established RAW264.7 cells stably expressing mCherry-GNA12 (*Gna12*^*mCherry*^) (supplementary Fig. S1A). Confocal imaging indicated the presence of GNA12^mCherry^ in the cytosol, as well as on the plasma membrane and some intracellular vesicles (Fig. 1C). Previous studies have found that GNA12 localizes to the mitochondria in human umbilical vein endothelial cells (HUVECs) (Andreeva *et al*. 2008). To determine the cellular localization of GNA12, we used MitoTracker to stain the mitochondria in the* Gna12*^*mCherry*^ cells and found no colocalization between GNA12 and mitochondria (supplementary Fig. S1B). Instead, GNA12^mCherry^ overlapped with lysosomes stained with LysoTracker (supplementary Fig. S1B). Therefore, GNA12 mainly localizes to the cytosol, plasma membrane and lysosomes in macrophages.

### GNA12 inhibits C5a-induced migration in macrophages

**Figure 2:**
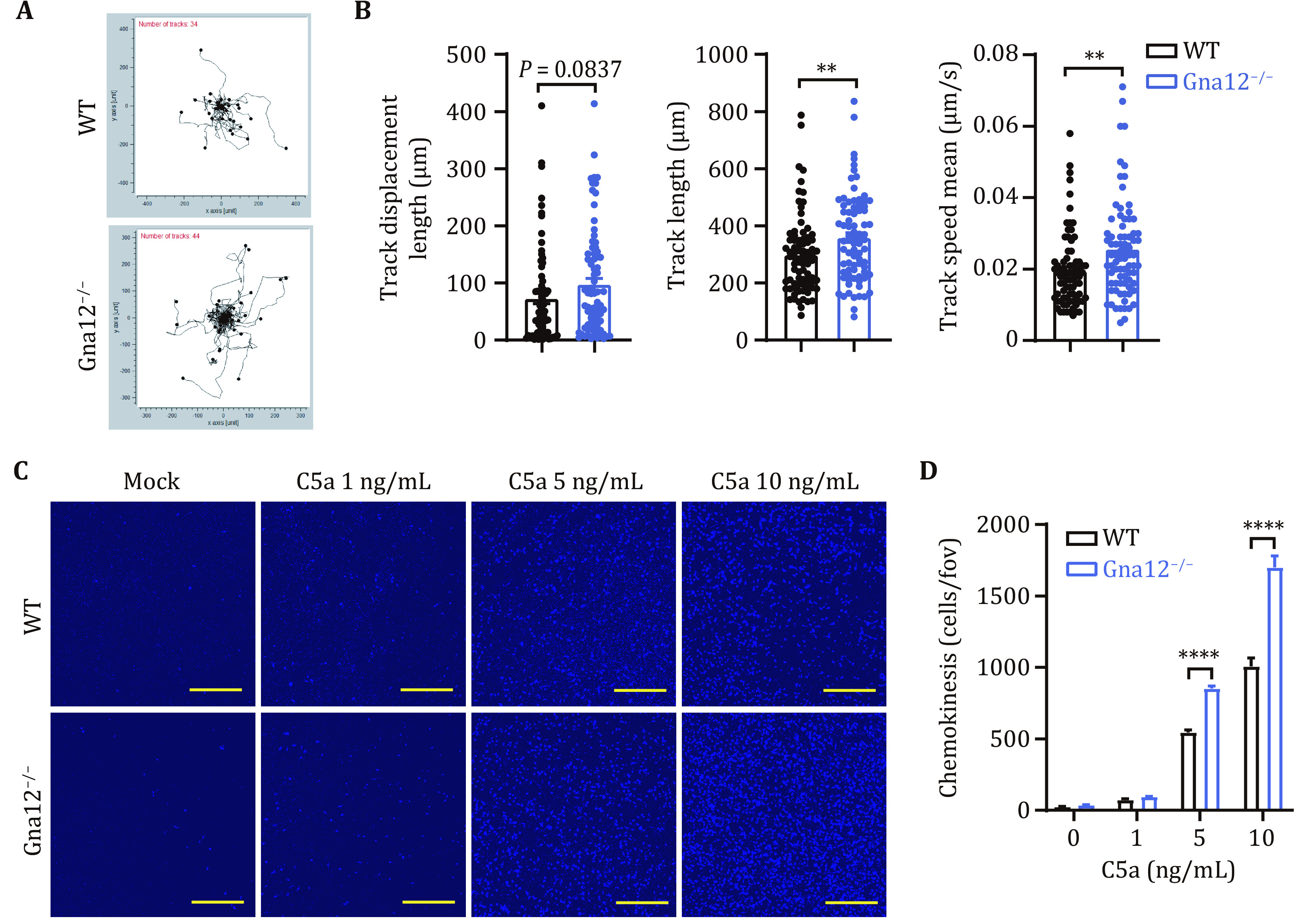
Deficiency of GNA12 enhances C5a-induced migration of BMDMs. **A** Migration trajectories of BMDMs from WT and *Gna12*^*-/-*^ mice at steady state. 34–44 cells were analyzed for each group. **B** Track displacement (left), track length (middle) and track speed (right) of WT and *Gna12*^*-/-*^ BMDMs. 80 cells were analyzed in each group. **C**,**D** The chemokinesis was evaluated with transwell migration assay between WT and *Gna12*^*-/-*^ BMDMs with C5a stimulation. Representative confocal images of migratory BMDMs stained with DAPI (blue). Scale bar, 500 μm (**C**). Number of cells migrated through the chamber (**D**). Data are expressed as mean and s.e.m. *P* values were calculated with Student’s *t*-test (**B**) and two-way ANOVA (**D**). **P <0.01; ****P <0.0001

We first determined the role of GNA12 in the movement of resting macrophages by tracking the movement of WT and *Gna12*^*-/-*^ BMDMs. Compared with WT BMDMs, *Gna12*^*-/-*^ BMDMs moved more freely, as evidenced by longer track lengths and faster speeds (Fig. 2A and 2B). We next examined the effect of GNA12 on C5a-induced migration. The migratory abilities of WT and *Gna12*^*-/-*^ BMDMs induced by C5a were assessed using a trans-well assay *in vitro*. At the presence of different concentrations of C5a, WT and *Gna12*^*-/-*^ BMDMs exhibited a dose-dependent migration (Fig. 2C and 2D). *Gna12*^*-/-*^ BMDMs exhibited higher migratory ability compared with WT BMDMs (Fig. 2C and 2D). Thus, GNA12 may play a negative role in regulating C5a-induced migration.

**Figure 3:**
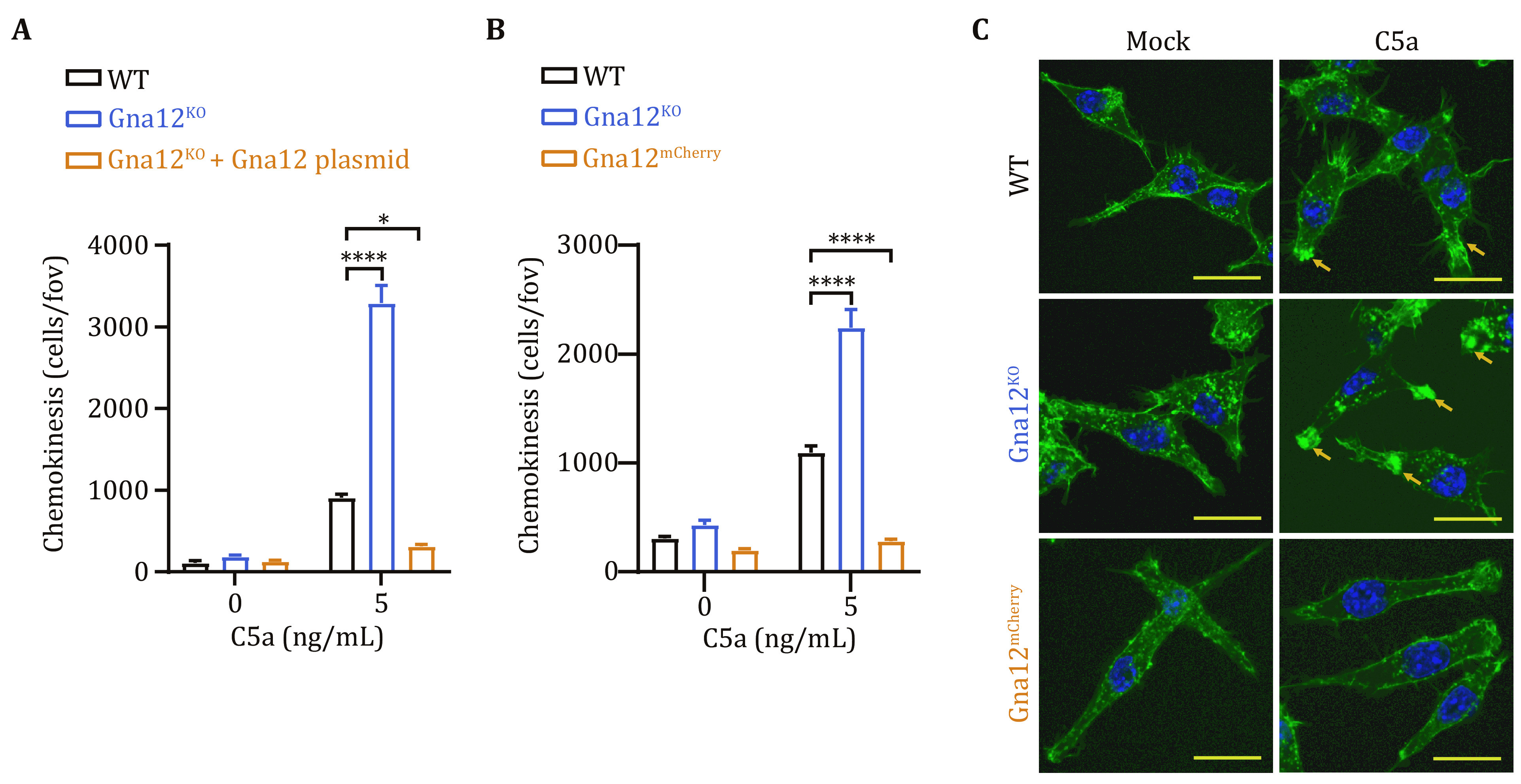
GNA12 inhibits C5a-induced migration of RAW264.7 cells. **A** The chemokinesis of WT, *Gna12*^*KO*^ and *Gna12*^*KO*^ RAW264.7 cells transfected with GNA12 was evaluated with transwell migration assay. **B** The chemokinesis of WT, *Gna12*^*KO*^ and *Gna12*^*mCherry*^ RAW264.7 cells was evaluated with transwell migration assay. **C** Confocal images of F-actin (green) in WT, *Gna12*^*KO*^ and *Gna12*^*mCherry*^ RAW264.7 cells. Nuclei were counterstained with DAPI (blue). Assemblies of F-actin at pseudopodia were marked with arrows. Scale bar, 20 μm. Data are expressed as mean and s.e.m. *P* values were calculated with two-way ANOVA (**A**, **B**). *P <0.05; ****P <0.0001

RAW264.7 cells stably deficient of GNA12 (*Gna12*^*KO*^), displayed enhanced migration induced by C5a (Fig. 3A, supplementary Fig. S2). Overexpression of GNA12 suppressed the enhanced migration in *Gna12*^*KO*^ (Fig. 3A). Conversely, we found that the GNA12-overexpressing (*Gna12*^*mCherry*^) cells displayed diminished migration induced by C5a (Fig. 3B). During cell migration, stress fibers are formed by polymerizing fibrous actin (F-actin) in pseudopodia, which provides power and support for cell migration (Akhshi *et al*. 2014). F-actin was stained with phalloidin in RAW264.7 cells either overexpressing GNA12 or knockout of GNA12, under the stimulation of C5a. There was a significant increase of F-actin in the pseudopodia of *Gna12*^*KO*^ cells, whereas the F-actin almost disappeared in *Gna12*^*mCherry*^ cells (Fig. 3C). Thus, GNA12 negatively regulates C5a-induced migration in macrophages.

### GNA12 regulates C5a-induced migration in macrophages by inhibiting the C5aR1-PLCβ2-PI3K-AKT-ERK1/2 pathway

**Figure 4:**
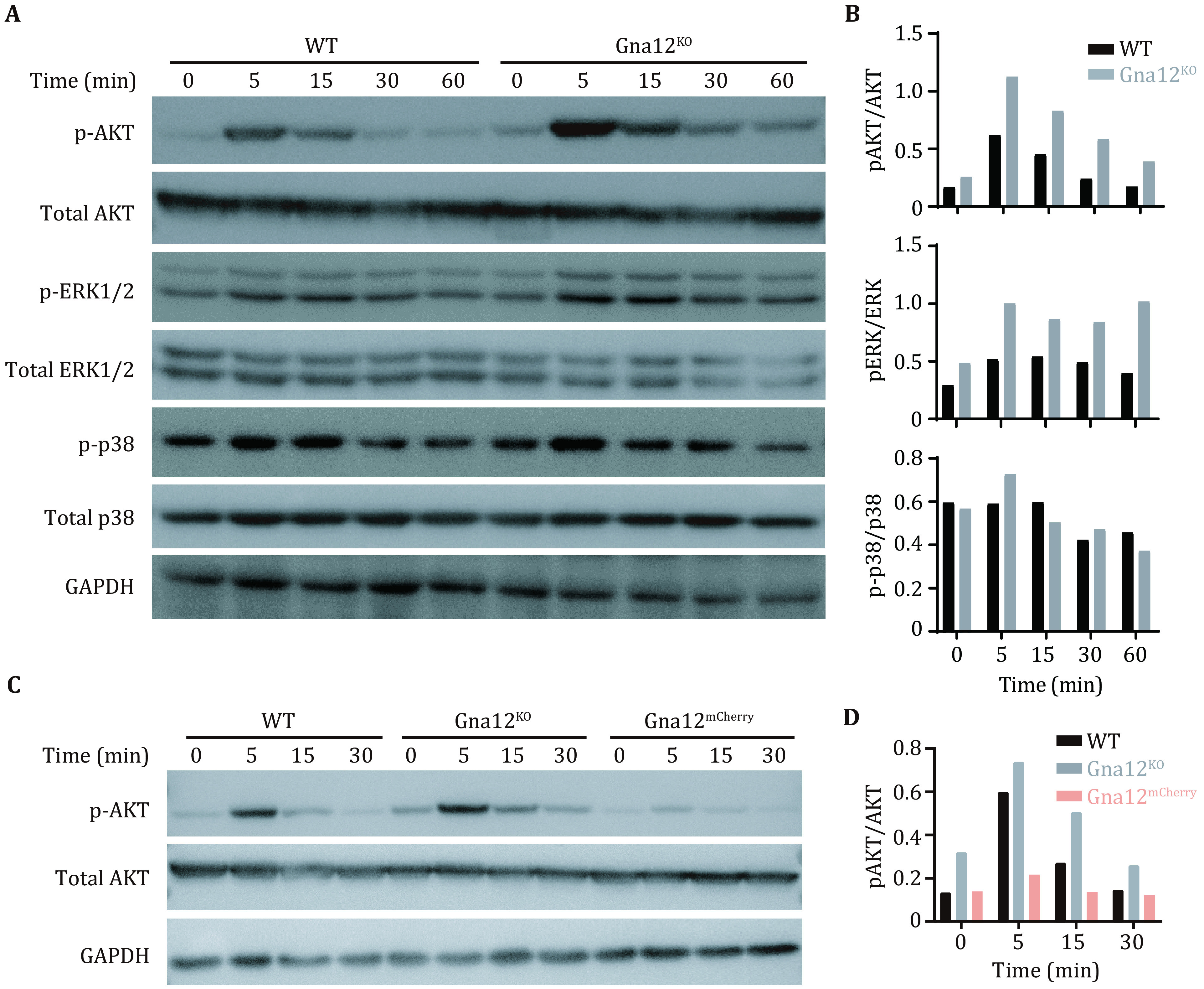
GNA12 inhibits the phosphorylation of AKT and ERK1/2 in RAW264.7 cells induced by C5a. **A** The levels of phosphorylated AKT, phosphorylated ERK1/2 and phosphorylated p38 were determined by immunoblotting of lysates from WT and* Gna12*^*KO*^ RAW264.7 cells treated with 10 ng/mL C5a for the indicated times. GAPDH was used as a loading control. **B** Grayscale statistics of AKT (upper), ERK1/2 (middle), and p38 (bottom) in Panel A. **C** The levels of phosphorylated AKT were determined by immunoblotting of lysates from WT, *Gna12*^*KO*^ and *Gna12*^*mCherry*^ RAW264.7 cells treated with 10 ng/mL C5a for the indicated times. GAPDH was used as a loading control. **D** Grayscale statistics of AKT in Panel C

After binding to C5a, C5aR1 activates multiple signaling proteins, including ERK and PI3K (Coffer *et al*. 1998; Monsinjon *et al*. 2003). It has been demonstrated that C5a induces the migration of macrophages through C5aR1-PLCβ2-PI3K-AKT-ERK1/2 and C5aR1-p38 signaling pathways (Chiou *et al*. 2004). We were prompted to examine whether GNA12 was involved in these two signaling pathways. The activation of these two pathways could be assessed by monitoring the phosphorylation level of the AKT, ERK1/2 and p38. We stimulated WT and *Gna12*^*KO*^ RAW264.7 cells with C5a. The phosphorylation of AKT and ERK1/2 were markedly enhanced in *Gna12*^*KO*^ RAW264.7 cells, compared with that in WT RAW264.7 cells (Fig. 4A and 4B). The phosphorylation level of p38 in *Gna12*^*KO*^ RAW264.7 cells was not significantly different from that in WT RAW264.7 cells (Fig. 4A and 4B). These data suggest that GNA12 deficiency enhanced the phosphorylation of AKT and ERK1/2 in response to C5a in macrophages. Conversely, overexpression of GNA12 suppressed activation of AKT in response to C5a in macrophages (Fig. 4C and 4D).

**Figure 5:**
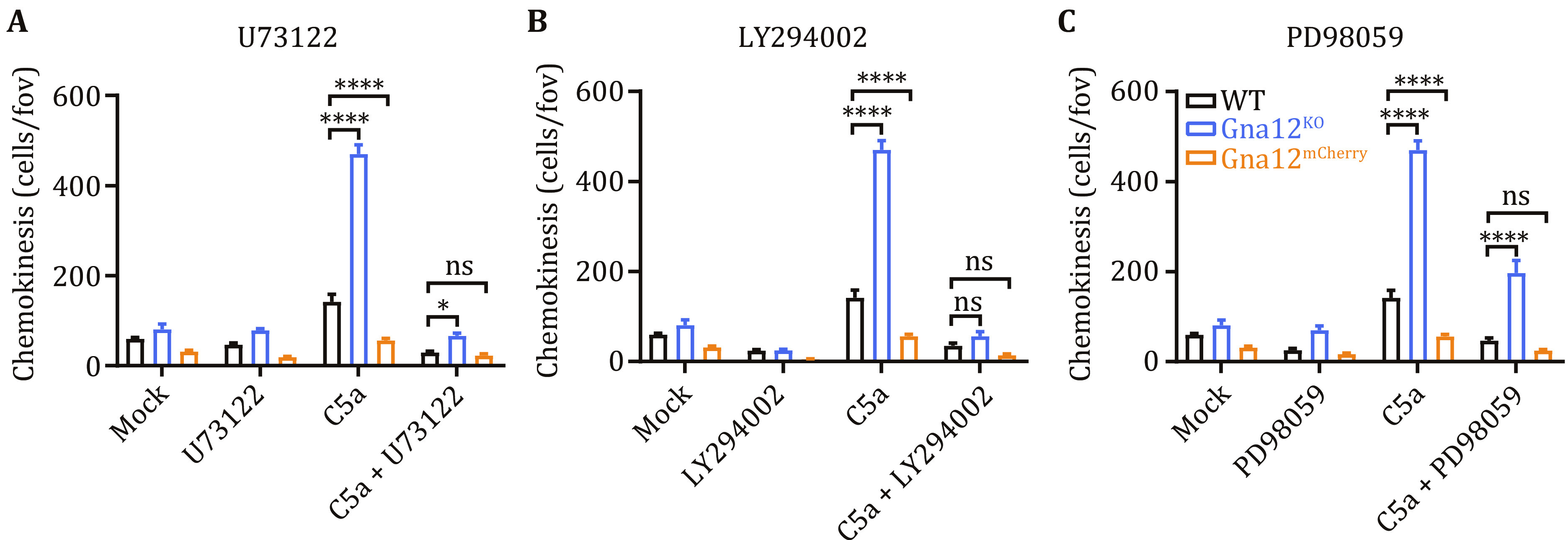
PLC, PI3K/AKT and ERK1/2 inhibitors significantly impair C5a-induced excessive migration in *Gna12*^*KO*^ RAW264.7 cells. **A**–**C** The effects of PLC inhibitor U73122 (**A**), PI3K/AKT inhibitor LY294002 (**B**) or ERK1/2 inhibitor PD98059 (**C**) on chemokinesis of WT, *Gna12*^*KO*^ and *Gna12*^*mCherry*^ RAW264.7 cells. The chemokinesis was evaluated with a transwell migration assay. Data are expressed as mean and s.e.m. *P* values were calculated with two-way ANOVA (**A**–**C**). *P <0.05; ****P <0.0001; ns, *P* >0.05

Next, we wondered whether modulating the C5aR1-PLCβ2-PI3K-AKT-ERK1/2 signaling pathway might suppress the enhanced migration caused by GNA12 deficiency. Treatments of U73122 (PLC inhibitor), LY294002 (PI3K/AKT inhibitor) and PD98059 (ERK1/2 inhibitor) all suppressed the enhanced migration in *Gna12*^*KO*^ RAW264.7 cells (Fig. 5). These results are consistent with our hypothesis that GNA12 inhibits C5a-induced migration in macrophages by suppressing the C5aR1-PLCβ2-PI3K-AKT-ERK1/2 signaling pathway.

### GNA12 interacts with C5aR1 and PLCβ2

**Figure 6:**
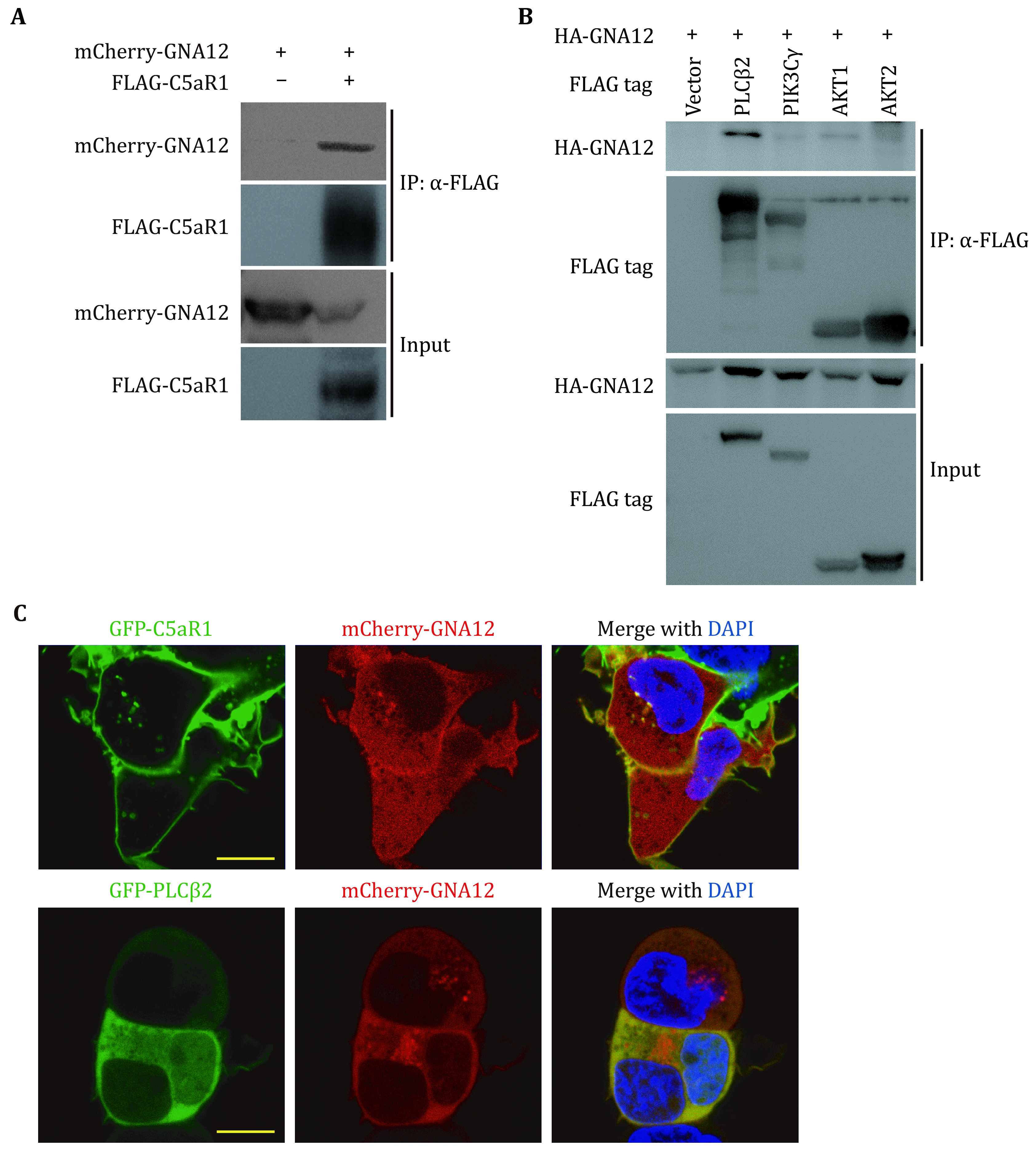
GNA12 interacts with C5aR1 and PLCβ2. **A** Co-immunoprecipitation (IP) analysis of the interaction between overexpressed GNA12 and C5aR1. **B** co-IP analysis of the interaction of GNA12 with PLCβ2, PIK3CG, AKT1 and AKT2. Tagged proteins were overexpressed in HEK293T cells, IP with anti-FLAG (**A**, **B**) beads and immunoblotted. **C** Colocalization of GNA12 and C5aR1 or PLCβ2 was detected by confocal microscope. HEK293T cells were transfected with mCherry-Gna12 plasmid and GFP-C5ar1 (upper) or GFP-Plcb2 (bottom) plasmid. Nuclei were counterstained with Hoechst (blue). Scale bar, 10 μm

To determine how GNA12 might regulate the C5aR1-PLCβ2-PI3K-AKT-ERK1/2 pathway, we examined the interactions between GNA12 and a series of proteins in this pathway. Co-immunoprecipitation revealed that GNA12 interacted with C5aR1 and PLCβ2, but not PIK3Cγ, AKT1 and AKT2 (Fig. 6A, 6B). Confocal imaging showed that C5aR1 predominantly localized to the cell membrane, while PLCβ2 localized to the cytoplasm (supplementary Fig. S3). We wondered whether GNA12 might colocalize with C5aR1 or PLCβ2. To test this, GFP-C5aR1 and mCherry-GNA12, or GFP-PLCβ2 and mCherry-GNA12 were overexpressed in HEK293T cells. Colocalization was observed on the plasma membrane between GFP-C5aR1 and mCherry-GNA12 (Fig. 6C). GFP-PLCβ2 was distributed in cytosol and overlapped with cytosolic mCherry-GNA12 (Fig. 6C). Thus, we suspected that GNA12 might interact with C5aR1 and PLCβ2 in regulating cell migration in macrophages.

## DISCUSSION

In this study, we uncover a role of GNA12 in regulating the chemotactic migration in macrophages. It is known that GNA12 and GNA13 collectively regulate C5a-induced migration (van den Bos *et al*. 2020). However, whether GNA12 alone is sufficient to regulate C5a-induced migration was unknown. Here, we demonstrate that GNA12 suppresses C5a-induced migration by downregulating the C5aR1-PLCβ2-PI3K-AKT-ERK1/2 signaling.

C5a recruits C5aR1-expressing monocytes and neutrophils to inflammation sites. Our study shows that GNA12 and C5aR1 colocalized on the plasma membrane, and co-immunoprecipitation data suggest that GNA12 interacted with C5aR1. Previous studies have shown that C5aR1 is down-regulated in activated neutrophils (Guo *et al*. 2003; Van Epps *et al*. 1993). After extended exposure to the C5a, the C5aR1 mainly co-localizes with the late endosomal/lysosomal marker LAMP2, and a fraction of C5aR1 is degraded in lysosomes (Suvorova *et al*. 2005). The down-regulation of C5aR1 results in reduced chemotaxis toward C5a in both CHO cells and neutrophils (Suvorova *et al*. 2005). Our data show that GNA12 is also localized to lysosomes. It is possible that GNA12 may affect the intracellular trafficking of C5aR1, which warrants future investigation.

Our study shows that GNA12 regulated C5a-induced migration in macrophages by inhibiting the C5aR1-PLCβ2-PI3K-AKT-ERK1/2 pathway. GNA12 and GNA13 belong to G_12_ subfamily in Gα family. G_12_ subfamily usually acts downstream of receptors via regulating the small GTPase Rho (Gadepalli *et al*. 2013; Mu *et al*. 2018; Ward *et al*. 2011). It has been shown that C5aR1 activates G_i_ protein and results in phospholipase C (PLC) activation (Chiou *et al*. 2004; Jiang *et al*. 1996; Klinker *et al*. 1996). Here, we show a negative role of GNA12 downstream of C5aR1. Indeed, Kyoto Encyclopedia of Genes and Genomes (KEGG) analyses indicated that the PI3K/AKT signaling is one of the major pathways defined as GNA12-dependent genes in SKOV3 cells (Ha *et al*. 2021). Thus, different subfamilies of Gα proteins may have opposite effects on the downstream signal transduction of the same signal.

PLCβ proteins are important for chemoattractant-mediated signal transduction (Li *et al*. 2000). Moreover, PLCβ has been shown to be a target of Gα_q_, which regulates intracellular Ca^2 + ^ and activation of protein kinase C (Dowal *et al*. 2006; Rebecchi and Pentyala 2000). Thus, Gα plays the intermediate role between GPCRs and PLCβ by interacting with them. PLCβ2 is involved in C5a-induced migration in macrophages (Chiou *et al*. 2004). Our data show that treatment of U73122 (a PLC antagonist) suppressed the enhanced migration in *Gna12*^*KO*^ RAW264.7 cell, indicating the dependence of PLCβ2. The co-IP experiment shows that GNA12 interacts with C5aR1 and PLCβ2. Thus, we believe that GNA12 interacts with C5aR1 and PLCβ2 to relay the signal. However, our results cannot exclude the possibility of indirect interaction between GNA12 and C5aR1 or PLCβ2, and the exact molecular mechanisms by which GNA12 regulates the signal transduction of C5aR1-PLCβ2 remain to be investigated.

GNA12 has been reported to mediate cell migration in different cell types, but the specific effect on cell migration depends on cell type and stimuli. For example, GNA12 together with GNA13 promotes gastrin-induced directional migration of pancreatic cancer cells via the cholecystokinin B receptor-GNA12/13-RhoA-ROCK signaling pathway (Mu *et al*. 2018). LPA promotes the interaction of GNA12 and EFA6 through LPAR2 and activates the ADP-ribosylation factors 6-based pathway, consequently promoting the invasion of renal cancer cells (Hashimoto *et al*. 2016). Thrombin induces the migration of monocytes via the PAR1-GNA12 pathway (Gadepalli *et al*. 2013). These studies demonstrate a role of GNA12 in promoting cell migration. On the contrary, knockout of GNA12 and GNA13 in macrophages results in increased cell velocity, but intact chemotaxis (van den Bos *et al*. 2020), indicating a role of GNA12 and GNA13 in inhibiting cell migration. Our data indicate a negative role of GNA12 in regulating C5a-induced migration. Collectively, GNA12 regulates cell migration in a manner dependent on cell type and stimuli.

Human genetic studies have indicated the involvement of *Gna12* in IBD. Previous studies show that the activation of GNA12 leads to phosphorylation of the tight junction proteins ZO-1 and ZO-2, resulting in the destabilization of cell junctions and increased paracellular permeability in epithelial cell lines (Sabath *et al*. 2008). It has been suspected that the role of GNA12 in regulating cell junctions may explain its involvement in IBD susceptibility. We noticed that *Gna12* is mainly expressed in BMDMs and peritoneal macrophages (data from BIOGPS). A large number of immune cells infiltrate the intestinal mucosa during colitis, of which macrophages are important players (Platt *et al*. 2010). C5a and C5aR1 have been shown to promote colitis, and pharmacological inhibition of C5a activity by PMX205 is efficacious in preventing DSS-induced colitis in mice (Jain *et al*. 2013). Our study demonstrates that GNA12 downregulates C5a-induced migration in macrophages. We propose that GNA12 is an anti-inflammatory factor, which might alleviate the development of inflammation by inhibiting the excessive chemotactic migration of macrophages. Whether GNA12 in intestinal macrophages affects the development of colitis through regulating C5a-induced cell migration remains to be explored.

## MATERIALS AND METHODS

### Mice

*Gna12*^*-/-*^ mice were generated using the CRISPR/Cas9 technique. 8–12-week-old gender-matched mice were used in the study. Mice were housed at 2–5 animals per cage in a 12-h light/12-h dark cycle with *ad libitum* access to food and water at a controlled temperature (23 ± 2 °C). All SPF mice, including wild-type C57BL/J6, were bred and housed in a barrier facility accredited by the Association for Assessment and Accreditation of Laboratory Animal Care International for SPF mice (Laboratory Animal Facility of Tsinghua University). All animal protocols were approved by the Committee of Institutional Animal Care and Research.

### Generation of *Gna12*^*-/-*^ mice with the CRISPR/Cas9 technique

sgRNA oligos targeting *Gna12* were designed using the online CRISPR design tool (http://crispr.mit.edu). Two partially complementary oligonucleotides (5'-CACCGGCGGACGTGCTCATATTCG-3' and 5'-AAACCGAATATGAGCACGTCCGCC-3') were synthesized (Invitrogen) and used to construct sgRNA-expressing vectors. Both the sgRNAs and the Cas9 mRNA were prepared and purified as described (Zhang *et al*. 2019).

*Gna12* mutant mice were generated by injection of *in vitro* transcribed sgRNAs (50 ng/μL) and Cas9 mRNA (100 ng/μL) into the cytoplasm of pronuclear stage C57BL/6J zygotes (Laboratory Animal Research Center, Tsinghua University). Approximately 100 zygotes were injected and subsequently transferred to the oviduct of pseudo-pregnant B6CBAF1 females, from which viable founder mice were obtained. The target sites of the viable founder mice were amplified by PCR with specific primer pairs (5'-CGTGTCCAGCCCTAACACCCTATTT-3' and 5'-CTCAGCAGTCCA ATAAGAAGCTCCC-3') and sequenced. *Gna12* mutant founders were identified.

### Cell culture and reagents

HEK293T cells and RAW264.7 cells were cultured in DMEM supplemented with 10% FBS and 1% penicillin/streptomycin at 37 °C in 5% CO_2_ in a humidified incubator.

BMDMs were cultured as described (Liu *et al*. 2011). Briefly, bone marrow cells from the femurs of mice were seeded into plates in DMEM supplemented with 10% FBS, 1% penicillin/streptomycin, and 20 ng/mL M-CSF (Novoprotein). After three days, fresh medium was added to the plate. The cells were ready for use on day 6.

### Generation of *Gna12*^*KO*^ RAW264.7 cell line

The *Gna12*^*KO*^ RAW264.7 cell line was generated by using the CRISPR-Cas9 System (Cas9-2hitKO). Two gRNAs (gRNA1, 5'-GACCTTTTTTGTACAACCGA-3'; gRNA2, 5'-TTTGCACACTGTGG CCCGAT-3') were designed to target to upstream of *Gna12* exon 3 and downstream of *Gna12* exon 4, respectively. gRNA oligos were designed using the online CRISPR design tool (http://crispr.mit.edu). The designed gRNAs were cloned into the PX458 and EZ-GuideXH plasmid, respectively. Then, the EZ-GuideXH-gRNA2 plasmid was cut by HindIII and XhoI, and the gRNA2 was cloned into the HindIII and XhoI sites of PX458-gRNA1 plasmid. Finally, this bigger plasmid was transfected by FuGENE HD (Promega) into RAW264.7 cells. The individual GFP^ + ^ cells were sorted into 96-well plates by BD FACS AriaII at 48 h post-transfection. Cell clones were cultured and expanded. PCR and immunoblotting were used to validate the clones. Three PCR primers (P1, 5'-GTATCACCGCACCTGGGTT-3'; P2, 5'-GGCCAATCCGGTCCAAGTTAT-3'; P3, 5'-AGAGCATCTCTGGTCCACCT-3') were designed. The P1 and P2 amplified WT band (564 bp), while the P1 and P3 amplified KO band (421 bp).

### Generation of *Gna12*^*mCherry*^ RAW264.7 cell line

To generate the* Gna12*^*mCherry*^ RAW264.7 cell line. The cDNA of *Gna12* was cloned into the pB-mCherry plasmid. pB-mCherry-Gna12 plasmid and pBase plasmid (3:1 ratio) were co-transfected into RAW264.7 cells. Transfected cells were selected with 700 μg/mL G418 for 14 days after 48 h of transfection. Then, the individual mCherry^ + ^ cells were sorted into 96-well plates by BD FACS AriaII. Cell clones were cultured and expanded, and immunoblotting was used to validate the clones.

### Treatment of RAW264.7 cells

RAW264.7 cells were treated with 10 ng/mL recombinant mouse C5a (Novoprotein, C075) for different periods of time before cells were harvested for immunoblotting. For phalloidin staining, RAW264.7 cells were seeded on the coverslips and cultured overnight in 24-well plates. Cells were treated with 10 ng/mL recombinant mouse C5a for 30 min before fixation and staining. For inhibitor treatment, RAW264.7 cells were treated with 25 μmol/L LY294002, 10 μmol/L PD98059 or 100 nmol/L U73122 for 30 min before transwell assay.

### Live cell staining

Cells were seeded in a glass bottom dish (JingAn Biological). LysoTracker Deep Red (Invitrogen) or MitoTracker Green (Invitrogen) were diluted in the cell culture medium according to the manufacturer’s guidance. Cells were incubated with the staining solution at 37 °C for 1 h. Then, cells were counterstained with Hoechst 33342 (Beyotime Biotechnology). Images were obtained with Nikon A1R confocal.

### Plasmids

Murine *Gna12* cDNA was reverse transcribed from mRNA prepared from murine BMDMs and cloned into the pmCherry-N1 and the pECMV-HA vector, respectively. Sequences were confirmed by Sanger sequencing. pECMV-Pik3cg-m-FLAG, pECMV-Akt1-m-FLAG, pECMV-Akt2-m-FLAG, pECMV-Plcb2-m-FLAG and pECMV-C5ar1-m-FLAG were purchased from Miaolingbio.

### Antibodies

The following antibodies were used: anti-GNA12 (Santa Cruz, sc-515445), anti-GAPDH (Huaxingbio, HX1832), anti-AKT (CST, 4691S), anti-p-AKT (CST, 4060S), anti-ERK1/2 (CST, 4695T), anti-p-ERK1/2 (CST, 4370T), anti-p38 (CST, 8690T), anti-p-p38 (CST, 4511T), anti-FLAG (BBI, D110005), anti-HA (Huaxingbio, HX1803), anti-mCherry (Abcam, ab12509), Goat anti-Mouse IgG (H + L)-HRP (MBL, 330), Mouse anti-Rabbit IgG-HRP (SouthernBiotech, 4090-05).

### Immunoblotting

Cells were harvested and lysed with pre-chilled RIPA buffer containing protease inhibitor cocktail (Roche) and phosphatase inhibitor cocktail (Roche) on ice for 30 min. Supernatants were obtained by centrifugation at 12,000 r/min for 10 min at 4 °C. Protein samples were separated on SDS-PAGE and then blotted onto PVDF membranes. Nonspecific binding sites were blocked by incubating the membrane in TBS-T (0.1% Tween 20) with 5% bovine serum albumin (BSA) for 1 h at room temperature. The membrane was then incubated with primary antibodies at 4 °C overnight. Next, the membrane was incubated with the secondary HRP-conjugated antibodies for 1 h at room temperature and visualized by using ECL detection reagent. Protein band intensity was quantified using ImageJ software.

### Co-immunoprecipitation (IP)

5 × 10^6^ transfected HEK293T cells were harvested, washed with 1 mL cold PBS, and lysed in 400 μL cold IP buffer (50 mmol/L Tris, 150 mmol/L NaCl, 2 mmol/L EDTA, 1% NP-40, 0.5 mmol/L PMSF, protease inhibitor cocktail) on ice for 30 min. Then supernatants were obtained by centrifugation at 12,000 r/min for 10 min at 4 °C. Supernatants (10 μL) were saved for input. The rest of the supernatants were incubated with 15 μL pre-washed anti-FLAG M2 affinity gel (sigma, A2220) by rotating at 4 °C overnight. The beads were washed three times with 400 μL IP buffer before being eluted with 30 μL SDS sample buffer (1× ). Inputs and eluents were subjected to immunoblotting analysis.

### Tracking assay

BMDMs were seeded in a glass bottom dish (JingAn Biological) and stained with WGA 555 (Invitrogen). Cells were automatically imaged over time using a Nikon A1R confocal microscope at multiple positions per well using a 10× air objective. Images were taken every 8 min, and a total of 36 images were taken. The track displacement length, track length and track speed mean of individual cells were calculated by using Imaris software. The migration trajectories were analysed by Chemotaxis and Migration Tool (Ibidi).

### Migration assay

Cell migration was assessed using a 6.5 mm Transwell with a 5.0 μm pore polycarbonate membrane insert. 1 × 10^5^ cells were placed inside the insert, and C5a (1–10 ng/mL) was added to the lower well of the chamber to assess the chemotactic activity. Then the entire chamber was incubated at 37 °C to initiate migration (2 h for BMDMs and 8 h for RAW264.7 cells). Non-migrated cells were wiped off with a cotton swab and then the filter was fixed and stained with DAPI (Invitrogen) to define the cell nuclei. Images were obtained with Nikon A1R confocal microscope. Chemokinesis was assessed by counting the number of migrated cells in 3–5 random fields (10× air objective) per well.

### Phalloidin staining

Cells grown on coverslips were washed with cold PBS, fixed in 4% PFA for 10 min at room temperature, then washed with cold PBS for three times. Actin Tracker-488 (Beyotime Biotechnology) was diluted 1∶100 in PBS containing 0.1% Triton X-100 to prepare the staining solution. 50 μL of the staining solution was added to each coverslip and incubated for 1 h at room temperature. Coverslips were then counterstained with DAPI and mounted on slides in Fluoromount-G (SouthernBiotech). Images were obtained with a Nikon A1R confocal microscope.

### Statistical analysis

All statistical analyses were conducted with GraphPad Prism version 8.0. For comparisons between two groups, significance was determined using the Student’s *t*-test. For comparisons among more than two groups, significance was determined using two-way ANOVA.

## Abbreviation

BMDMs　Bone marrow-derived macrophages

C5a　　 Complement component 5a

CD　　　 Crohn’s disease

DLBCL　 Diffuse large cell B-cell lymphomas

F-actin　 Fibrous actin

GWAS　 Genome-wide association studies

GNA12 Guanine nucleotide-binding protein subunit alpha-12

HUVECs　Human umbilical vein endothelial cells

IBD　 Inflammatory bowel disease

KEGG　 Kyoto encyclopedia of genes and genomes

UC　 Ulcerative colitis

## Conflict of interest

Haonan Yu and Zhihua Liu declare that they have no conflict of interest.
